# Clinical trial readiness to solve barriers to drug development in FSHD (ReSolve): protocol of a large, international, multi-center prospective study

**DOI:** 10.1186/s12883-019-1452-x

**Published:** 2019-09-10

**Authors:** Samantha LoRusso, Nicholas E. Johnson, Michael P. McDermott, Katy Eichinger, Russell J. Butterfield, Elena Carraro, Kiley Higgs, Leann Lewis, Karlien Mul, Sabrina Sacconi, Valeria A. Sansone, Perry Shieh, Baziel van Engelen, Kathryn Wagner, Leo Wang, Jeffrey M. Statland, Rabi Tawil, on behalf of the ReSolve Investigators and the FSHD CTRN18, Mazen Dimachkie, Mamatha Pasnoor, Kiley Higgs, Katherine Roath, Ayla McCalley, Melissa Currence, Laura Herbelin, Rabi Tawil, Johanna Hamel, Leann Lewis, Katy Eichinger, Samantha LoRusso, W. David Arnold, Tabitha Alexander, Matthew Yankie, Kristina Kelly, Nicholas Johnson, Brittney Holmberg, Liz Diaz, Aileen Jones, Amanda Butler, Russell J. Butterfield, Sarah Moldt, Amelia Wilson, Melissa McIntyre, Kathryn Wagner, Doris Leung, Genila Bibat, Mary Yep, Nikia Stinson, Andrea Jaworek, Leo Wang, Laura Sissons-Ross, Laura Johnstone, Perry Shieh, Christy Skura, Dianne DeGuzman, Karlien Mul, Yvonne Cornelissen, Sabrina Sacconi, Luisa Villa, Angela Puma, Manuela Gambella, Ying Shi, Jeremy Garcia, Valeria A. Sansone, Elena Carraro, Fatmira Beshiri, Luca Mauro

**Affiliations:** 1 Department of Neurology, Ohio State University Wexner Medical Center, 395 W. 12th Ave., 7th Floor, Columbus, OH 43210 USA; 2 Department of Neurology, Virginia Commonwealth University, 1101 East Marshall St, PO Box 980599, Richmond, VA 23298 USA; 3 Department of Biostatistics and Computational Biology and Department of Neurology, University of Rochester Medical Center, 265 Crittenden Blvd., CU 420630, Rochester, NY 14642 USA; 4 Department of Neurology, University of Rochester Medical Center, Box 673, 601 Elmwood Ave, Rochester, NY 14642 USA; 5 Department of Pediatrics and Neurology, University of Utah, Eccles Institute of Human Genetics, Room 2260A, 15 N 2030 E, Salt Lake City, UT 84112 USA; 6 The NEMO Clinical Center, Neurorehabilitation Unit, University of Milan, Piazza dell’Ospedale Maggiore, 3, Milan, 20162 Italy; 7 Department of Neurology, University of Kansas Medical Center, 3901 Rainbow Blvd, MS 2012, Kansas City, KS 66160 USA; 8 Department of Neurology, Radboud University Medical Center, Reinier Postlaan 4 (935), 6525 GC Nijmegen, The Netherlands; 9 Université Côte d’Azur, Peripheral Nervous System, Centre Hospitalier Universitaire de Nice, Muscle & ALS Department, Pasteur 2 Hospital, 30 Voie Romaine, 06001 Nice Cedex 1, France; 10 Department of Neurology, University of California, Los Angeles, 300 Medical Plaza, Suite B-200, Los Angeles, CA 90095 USA; 11 Center for Genetic Muscle Disorders, Kennedy Krieger Institute, 707 N. Broadway, Baltimore, MD USA; 12 Department of Neurology, University of Washington, 1959 NE Pacific St, Seattle, WA 98195 USA

**Keywords:** Facioscapulohumeral muscular dystrophy, Muscular dystrophy, Outcome measures, Clinical trial, Functional testing, Electrical impedance Myography, Biomarkers

## Abstract

**Background:**

Facioscapulohumeral muscular dystrophy (FSHD) is a dominantly-inherited progressive muscular dystrophy caused by de-repression of the *DUX4* gene, which causes disease by a toxic-gain-of-function. As molecularly targeted drugs move from preclinical testing into human trials, it is essential that we validate clinical trial tools and methodology to facilitate the drug development process.

**Methods/design:**

The primary goal of this study is to hasten drug development for FSHD by validating two novel clinical outcome assessments (COAs) and refining clinical trial strategies. We will perform an 18-month longitudinal study in 220 genetically confirmed and clinically affected participants using our FSHD Clinical Trial Research Network, comprised of 8 sites in the United States, and 3 collaborating sites in Europe. Visits occur at baseline and months 3, 12, and 18. At each visit we will collect: 1) a novel FSHD functional composite COA made up of 18 evaluator-administered motor tasks in the domains of shoulder/arm, hand, core/abdominal, leg, and balance function; and 2) electrical impedance myography as a novel muscle quality biomarker (US sites). Other COAs include 1) Domain 1 of the Motor Function Measure; 2) Reachable workspace; 3) orofacial strength using the Iowa Oral Performance Instrument; 4) lean muscle mass using dual-energy X-ray absorptiometry (DEXA); 5) strength as measured by quantitative myometry and manual muscle testing; and 6) the FSHD Health Index and other patient-reported outcomes. Plasma, DNA, RNA, and serum will be collected for future biomarker studies. We will use an industry standard multi-site training plan. We will evaluate the test-retest reliability, validity, and sensitivity to disease progression, and minimal clinically important changes of our new COAs. We will assess associations between demographic and genetic factors and the rate of disease progression to inform refinement of eligibility criteria for future clinical trials.

**Discussion:**

To the best of our knowledge, this is the largest collaborative study of patients with FSHD performed in the US and Europe. The results of this study will enable more efficient clinical trial design. During the conduct of the study, relevant data will be made available for investigators or companies pursuing novel FSHD therapeutics.

**Trial registration:**

clinicaltrials.gov NCT03458832; Date of registration: 1/11/2018

**Electronic supplementary material:**

The online version of this article (10.1186/s12883-019-1452-x) contains supplementary material, which is available to authorized users.

## Background

Facioscapulohumeral muscular dystrophy (FSHD) is the second most common type of adult muscular dystrophy with an estimated prevalence range of 2–12 per 100,000 [1]. It is mostly dominantly inherited, but new mutations likely account for more than 10% of cases [2]. The disease is characterized by slowly progressive, asymmetric weakness that starts in the face and scapular muscles between ages 15–30 [3]. It later progresses to involve the truncal muscles and lower extremities, with about 20% of those affected eventually using a wheelchair [4]. However, there is a large degree of clinical variability in both disease progression and severity, even within families [3]. This makes predicting an individual’s disease course difficult and has made clinical trial design challenging.

The molecular defect in FSHD was discovered in 1992, and is located in the D4Z4 region on chromosome 4q35, a region with large repeated elements. The unified genetic model suggests two necessary requirements for the development of FSHD: 1) epigenetic de-repression of the D4Z4 region, either through contraction of the D4Z4 repetitive element (normal individuals > 10 repeats; FSHD 1–10 repeats), or through a second mutation in a gene involved in chromatin repression – both of which lower methylation and open the chromatin structure in this region; and 2) a permissive 4qA polymorphism that includes a polyadenylation sequence just distal to the last D4Z4 repeat. This results in de-repression of the Double Homeobox 4 (*DUX4*) gene, which is contained in the D4Z4 repeats and normally silenced in somatic cells [5]. *DUX4* causes disease by a toxic gain-of-function. While there are currently no effective pharmacological treatments [6], this genetic model has provided targets for drug development.

### Drug development landscape

The unusual FSHD disease mechanism, the epigenetic reactivation of the *DUX4* gene, is particularly amenable to knock-down of *DUX4* using epigenetic strategies or RNA therapies, as well as to other interventions targeting the downstream effects of *DUX4* expression. Current research involves the use of antisense oligonucleotide therapies (ASOs) to target inactivation of *DUX4*, and the success of ASOs for Duchenne muscular dystrophy (DMD) and spinal muscular atrophy provide a regulatory pathway for those chemistries [7, 8]. Exogenous siRNA targeting *DUX4* transcription and AAV-delivered RNA silencing of *DUX4* are also being investigated [9, 10], and some researchers are using screens of small molecules to identify drugs that affect D4Z4 methylation or translation of *DUX4* [11]. Along with molecular approaches to FSHD, non-specific therapies that may provide a benefit on muscle mass or function are also planned, and one placebo-controlled trial of an anti-myostatin therapy is currently underway (NCT02927080) [12].

### Clinical trial readiness

The increasing pace of drug development has created a pressing need for clinical trial preparedness. The importance of clinical trial planning is evident in the recent experience of DMD where an incomplete understanding of the 6 min walk test, the primary outcome variable in studies of premature stop and exon skipping therapies, led to inconclusive studies and costly delays [13–15]. This difficulty as well as problems standardizing measurements for biomarkers presented barriers as companies moved forward to try to gain FDA approval for accelerated access or marketing [16, 17].

Meetings with industry, advocacy groups, and FSHD researchers have identified several gaps in FSHD clinical trial readiness. Other than manual muscle testing (MMT) and quantitative myometry (QMT), there are no validated outcome measures used consistently in clinical trials in FSHD. A variety of individual functional measures have been investigated, and while most of these measures are reliable, none, individually, have been shown to be sensitive to disease progression over 1 year [18, 19]. A functional FSHD composite outcome measure (FSHD-COM) was recently developed and is novel in that the selected motor tasks reflect patient-reported significant domains of functional impairment [4, 20]. It has shown high test-retest reliability and strong cross-sectional associations with disease duration, clinical severity, and strength, but multi-site reliability and sensitivity to disease progression are yet to be demonstrated [21].

The identification and development of biomarkers will also be important for future clinical trials, especially to accelerate early phase trials. There are studies evaluating potential serum biomarkers but these are in early stages [22, 23]. Imaging and electrodiagnostic biomarkers are also being evaluated. Muscle MRI studies in FSHD have demonstrated fatty infiltration and structural changes that could be quantified and followed over time [24–27]. These structural changes demonstrated strong cross-sectional correlations with measures of strength and function but longitudinal studies assessing MRI changes over time are currently lacking [24–26]. Electrical impedance myography (EIM) is another tool that quantitatively and non-invasively measures changes in muscle composition. It uses subthreshold electrical current to determine the impedance to current flow through a particular muscle or muscle group. It has shown good reliability and strong associations with measures of disease severity in diverse neuromuscular diseases, including FSHD, spinal muscular atrophy, and DMD, and is responsive to disease progression in DMD and amyotrophic lateral sclerosis [28–32]. The potential advantages of EIM are readily apparent when collecting the measurements. It is painless, requires minimal training, and does not require specific expertise in post-processing. It was found to have good cross-sectional validity in FSHD [31]; however, a more recent study with 32 patients did not show sensitivity to disease progression over 12 months in a clinically relatively stable set of patients [33].

In addition to biomarker identification, there is a broad consensus that a better understanding of the relationships between genetic and clinical features and disease progression may be helpful for refining trial eligibility criteria. Previous cross-sectional studies have identified possible genetic and demographic correlates of disease progression. For instance, patients with the smallest number of residual D4Z4 repeats generally have more severe disease. These patients are typically diagnosed at a younger age, have higher penetrance by age [34], are more likely to use a wheelchair [4, 35], and are more likely to experience extra-muscular manifestations of FSHD [36–38]. However, no prospective study has determined whether such genetic differences have implications for disease progression over typical clinical trial time frames.

### Objectives

The overall aim of this study is to hasten drug development for FSHD. To this end we will further develop two novel clinical outcome assessments (COAs): the FSHD-COM and a skeletal muscle biomarker, EIM. In addition, we hope to gain a better understanding of the relationships between genetic and demographic features and disease progression, which will be valuable for refining eligibility criteria for future clinical trials. The specific objectives are:

- *Objective 1: Determine the Multi-Site Reliability and Validity of New COAs.* We hypothesize that both the FSHD-COM and EIM will be reliably measured at multiple sites and reflect disease severity. Having already established single site test-retest reliability and convergent validity by examining associations between the new COAs and other FSHD outcomes such as strength and lean muscle mass, we will confirm these findings in a large multi-site study.
- *Objective 2: Compare the Responsiveness of New COAs to Those of Other FSHD Outcomes and Determine the Minimal Clinically Important Changes (MCICs).* We hypothesize that our new COAs will be sensitive to disease progression. We will determine the responsiveness of our COAs to disease progression over 12 and 18 months and compare it to those of other FSHD outcomes, including strength and patient-reported motor disability. We will determine the variability in our COAs in a large cohort for sample size calculations to plan future trials. We will use a variety of techniques to determine the change in the FSHD-COM that would be of minimal clinical importance.
- *Objective 3: Establish FSHD Cohort Characteristics Useful for Determining Clinical Trial Eligibility Criteria.* We hypothesize that known genetic and demographic correlates of disease severity will account for some of the variability in disease progression, and can help refine trial eligibility criteria. Potential correlates of disease progression include D4Z4 residual fragment size, baseline functional status, age, and sex. We will implement statistical methods to determine specific subgroups of people with FSHD who are more or less likely to progress over 12–18 months and use this information to establish eligibility criteria for future clinical trials.
- *Objective 4: Collect biological samples for future biomarker studies.* While this study will not directly study tissue biomarkers, collecting serum, plasma, RNA, and DNA from a large number of carefully characterized FSHD patients followed for 18 months provides a unique opportunity to create an invaluable biorepository.

## Methods/design

### Study design

**Fig. 1:**
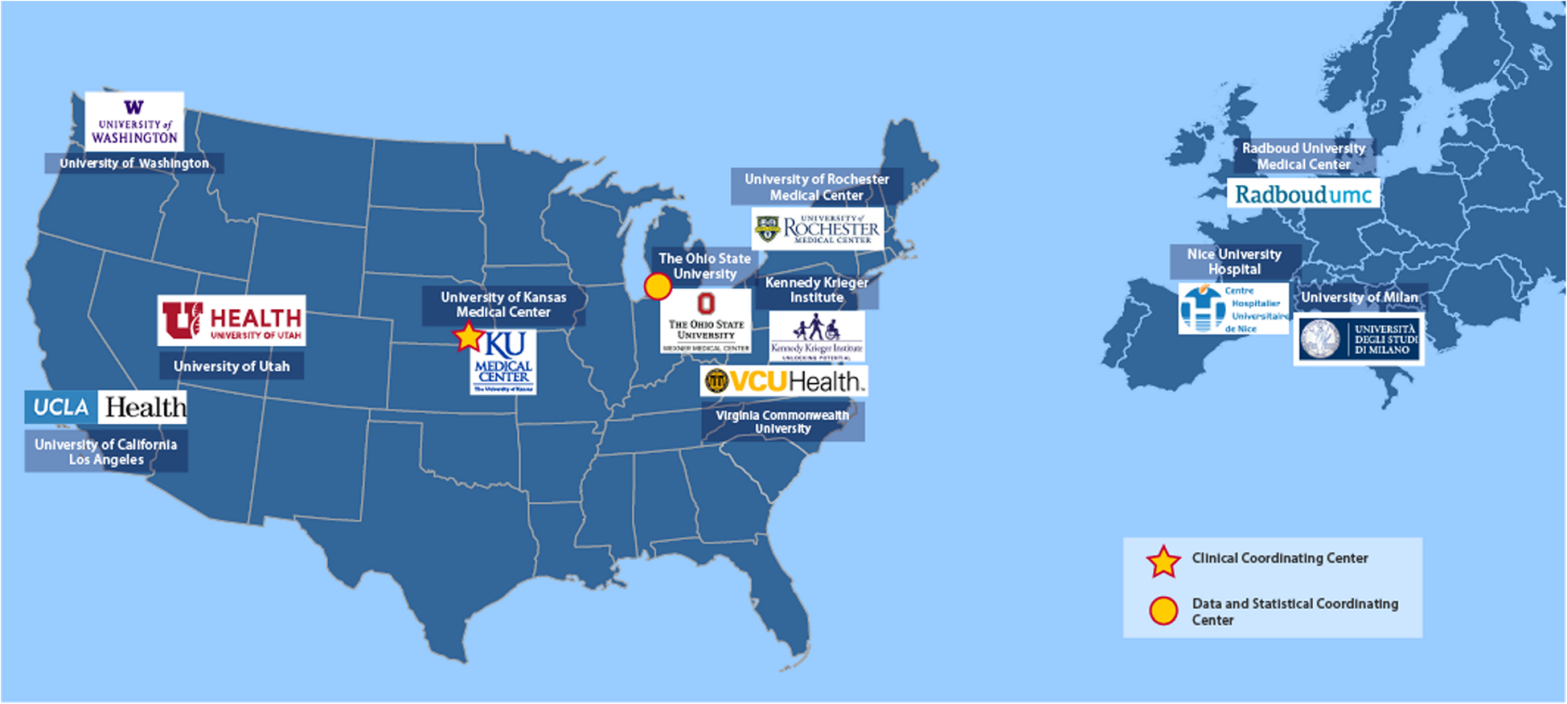
Title: The FSHD-CTRN Legend: The FSHD-CTRN is made up of 8 US sites, with the University of Kansas Medical Center serving as the central IRB and central coordinating center, and the University of Rochester serving as the data coordinating center. In addition, 3 collaborating sites in Europe are participating in the study, with the exception that they will not be performing EIM. Source: http://www.kumc.edu/fshd/our-sites.html

This is a prospective, multi-center, 18-month study of 220 FSHD patients. The study began recruitment in January, 2018 and the first patient was enrolled in March, 2018. Recruitment and data collection is ongoing and will continue until 220 patients are enrolled. An established FSHD Clinical Trial Research Network (FSHD-CTRN) [39], supported by the Muscular Dystrophy Association and the FSH Society, with experienced clinicians and clinical evaluators will be utilized to conduct the study (Fig. 1). The sites were chosen for their wide geographic distribution, experience in neuromuscular clinical research in general and FSHD in particular, prior collaborative work among the sites, and access to a large number of patients with FSHD.

### Protocol development and patient engagement

Collaboration with industry, advocacy groups, and FSHD researchers played an important role in the development of the current study. These meetings, which included two clinical trial preparedness workshops, helped to identify the major gaps that need to be addressed in order accelerate efficient drug development [40, 41]. In addition to seeking guidance from multiple sources prior to developing the protocol, collaboration and continued dialogue throughout the course of the study has been a priority.

We are using the FSHD-CTRN patient engagement model to address specific aims or difficulties encountered in running the proposed study; for example, defining what would be clinically meaningful to people with FSHD, addressing concerns related to participating in clinical studies, and issues with recruitment and retention. We hope that this strategy will help mitigate attrition bias. Specific feedback was sought from 2 to 4 individuals with FSHD at each CTRN site. Qualitative data were analyzed to further refine areas of functional importance to patients and the adequacy of functional measures included in the current study. This feedback was reviewed at the annual meeting. Continued feedback will be sought in this manner throughout the duration of the study.

### Multi-site training plan

A pre-study multi-site training meeting was convened in Year 1 to review the protocol and train clinical evaluators (CEs) and investigators. The CEs had a separate hands-on training session on all study procedures, and the Investigators had one for EIM training. At the training sessions, intra-rater reliability was determined by comparing performance on patient volunteers evaluated twice within a 24-h period. At wrap-up there was open discussion of challenges encountered in testing, discrepancies in results were discussed and resolved, and recommendations to improve reliability or efficiency of testing were adopted. Hands-on training was supplemented by access to videos demonstrating proper procedures for functional and strength testing and EIM. When new CEs or Investigators join the study, they will be trained by webinar on techniques, and then have in-person testing with the main CE (FSHD-COM) or closest CTRN investigator (EIM). Annual meetings will occur throughout the conduct of the study to review the protocol, review recruitment and challenges with achieving study goals, and refresh training on the FSHD-COM and EIM testing. Our plan to promote reliability of evaluation procedures is modeled after an industry approach where a trained lead Evaluator or Investigator trains site personnel to promote consistency of technique and identify areas requiring further training.

### Subject recruitment and consent

Study subjects will be recruited through a variety of methods which will help to lessen potential sampling bias. Subjects will be identified using local neuromuscular patient lists, national and international FSHD registries, electronic health record review at sites with Clinical Translational Science Awards (CTSAs), and registration of the study on clinicaltrials.gov. In order to reach traditionally under-represented populations, we will use local CTSA community outreach officers to set up meetings with under-represented communities and discuss the study and study goals.

During the process of consent, after all study procedures have been described and questions have been answered, and prior to signing the informed consent form, the subjects will be asked to describe their understanding of the study and their role in the study. The study is voluntary, and subjects will be made aware they can withdraw from the study at any time for any reason.

### Study population

The subject selection criteria are meant to yield the types of subjects likely to be selected for FSHD clinical trials; however, the criteria are still broad enough to investigate demographic and genetic factors that may be associated with disease progression.

#### Inclusion criteria

Age 18–75; genetically confirmed FSHD1 or a clinical diagnosis of FSHD with characteristic findings on exam and an affected parent or offspring [42]; symptomatic limb weakness; able to walk 30 ft without the support of another person (canes, walking sticks, and braces are allowed but not walkers); if taking over the counter supplements, willing to remain consistent with the regimen throughout the study.

#### Exclusion criteria

Cardiac or respiratory dysfunction that is deemed clinically unstable or would interfere with safe testing; orthopedic conditions that preclude safe testing of muscle function; regular use of muscle anabolic/catabolic agents such as corticosteroids, oral testosterone or oral beta agonists; use of an experimental drug in an FSHD clinical trial within the past 30 days; and pregnancy.

We have not included patients with FSHD2 as they represent only about 5% of the FSHD population and recruitment of a sufficient number of these patients to obtain meaningful results is not practical. It is possible that individuals may inadvertently be enrolled who will subsequently be found to have FSHD2, or an alternative diagnosis. However, the expectation is that this will be less than 5% of individuals without genetic confirmation prior to enrollment. If genetic testing performed as part of this study fails to confirm the genetic diagnosis, the results of the genetic test will be shared with the participant, and their participation in the study will end.

### Assessments

Enrolled subjects will be seen at the participating sites for a two-day evaluation at the first visit and a single day evaluation at each subsequent visit to include the schedule of activities outlined in Table 1. To establish intra-rater reliability at each site, all subjects will be reevaluated using the FSHD-COM, Reachable workspace, Iowa Oral Performance Instrument (IOPI), and EIM on Day 2 of Visit 1. In order to further characterize our cohort with respect to disease severity and evaluate the validity of the FSHD-COM and EIM, other evaluations will include MMT and QMT, dual energy X-ray absorptiometry (DEXA) lean muscle mass, and two commonly used FSHD clinical severity scales (The Clinical Severity Score [CSS] and the FSHD Clinical Score [FCS]) [43, 44]. Patient-reported outcome measures including the PROMIS57, the upper extremity functional index (UEFI), the facial disability index (FDI) physical score and the recently developed FSHD-Health Index (FSHD-HI) will also be completed [20, 45–50].

**Table 1:** Schedule of assessments

Visits	Visit 1		Visit 2	Visit 3	Visit 4
	Day 1	Day 2			
Time (Month)	0		3	12	18
			+/− 1 Week	+/− 2 Weeks	+/− 2 Weeks
Obtain Consent	X				
Confirm Eligibility	X				
Urine Pregnancy Test^a^	X		X	X	X
History and Physical Exam	X		X	X	X
Blood for FSHD DNA Testing	X		X		
Blood for Serum Extraction	X		X	X	X
Blood for RNA (PAX gene tube)	X		X		
Blood for Plasma (EDTA tube)	X		X	X	X
PROs: FSHD-HI, PROMIS57, UEFI, and FDI physical score	X		X		X
Electrical Impedance Myography	X	X	X	X	X
FSHD-COM / MFM Domain 1	X	X	X	X	X
Strength Testing: QMT and MMT	X		X	X	X
Bedside spirometry	X		X	X	X
DEXA	X			X	
Clinical Severity Scores	X		X	X	X
Iowa Oral Performance Instrument	X	X	X	X	X
Reachable Workspace	X	X	X	X	X
Fall/Exercise Questionnaire	X		X	X	X
Fall Assessment^b^			X		
“Domain-delta” questionnaire			X	X	

^a^Urine pregnancy test for women of childbearing age, ^b^ weekly × 12 weeks after the month 3 visit

Assessments occur at baseline, 3, 12, and 18 months. The FSHD-COM, electrical impedance myography, MFM Domain 1, Iowa Oral Performance Instrument, and Reachable Workspace are performed on Day 1 and Day 2 of the baseline visit in order to establish intra-rater reliability

### FSHD-COM

The FSHD-COM is an 18-item evaluator-administered instrument comprised of individually validated functional motor tasks (Table 2) [21]. The body regions represented match areas of importance identified by patients and include: leg function, shoulder and arm function, trunk function, hand function, and balance. The total scale has 72 points, with larger weight given to the most commonly patient-noted areas of functional concern – leg function and shoulder and arm function. Each individual item is scored from 0 to 4, with 4 representing the worst performance and 0 corresponding to unaffected/normal performance. While this composite measure was intended to evaluate ambulatory patients, it is still capable of capturing differences in functional performance for those that are non-ambulatory, thus avoiding any floor effects [21].

**Table 2:** The FSHD-COM

Region	ITEM	References
Leg function	Sit to stand without hands	[51–53]
	6 Minute Walk	[54–56]
	Self-selected gait speed	[57–59]
	Go 30′	[49, 55, 60]
	Ascend/ descend stairs	[49, 55, 60]
Shoulder / arm function	Shoulder Abduction (R/L)	[18, 60, 61]
	Shoulder Forward Flexion (R/L)	[18, 60, 61]
	Elbow Flexion (R/L)	[18, 60, 61]
	Don/doff Coat	[62]
Trunk function	Pick up a penny from floor	[62]
	Sit up with feet held	[4]
	Supine to sit	[4]
Hand function	Hand Grip Force Men	[49, 63–65]
	Hand Grip Force Women	[49, 63–65]
Balance	TUG: Timed up and Go	[66, 67]

The FSHD-COM is an 18-item instrument comprised of individually validated motor tasks. More weight is given to leg function and shoulder/arm function which are the two most frequently cited areas of patient concern (table modified from Eichinger et al. [21])

### Electrical Impedance Myography (EIM)

EIM testing is non-invasive and will be administered by a research study team member with proper training in using EIM to assess the muscles of individuals with neuromuscular disease. Prior studies have demonstrated the reliability, validity, and sensitivity of EIM in FSHD in a single site cohort [31, 33]. The tests are not considered to involve more than minimal risk. The estimated testing duration for an individual subject is 30 min.

**Fig. 2:**
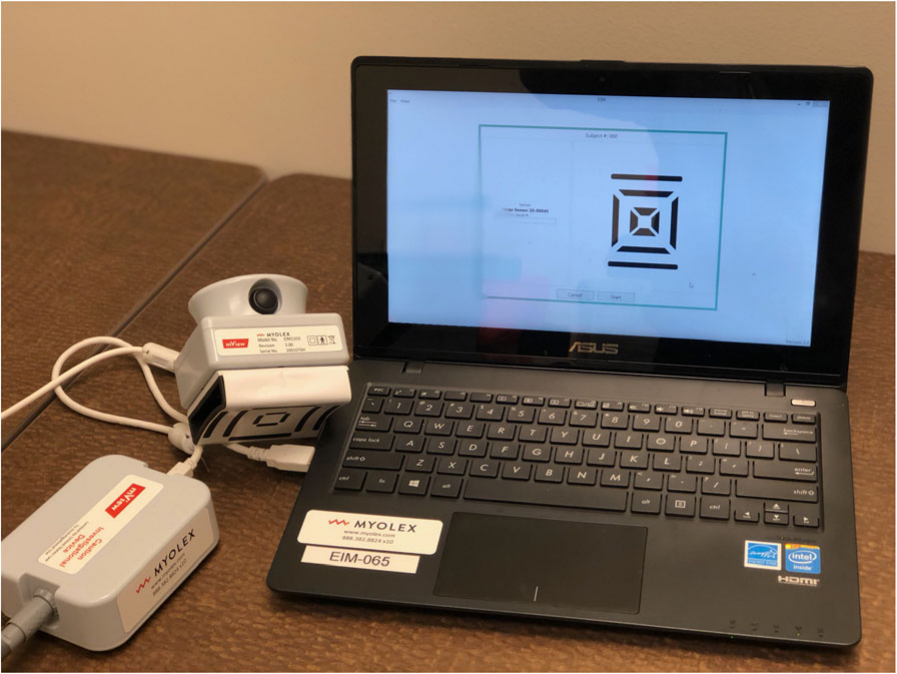
Electrical Impedance Myography Legend: The EIM device consists of a laptop computer connected to a portable handheld sensor

EIM is administered using an investigational device manufactured by Skulpt, Inc. (Boston, MA) that non-invasively measures the impedance of skeletal muscle over a frequency range between 1 kHz and 10 MHz (Fig. 2). The impedance is measured at each frequency by applying low-intensity electrical current (< 1 mA) via surface electrodes; the resulting voltage signals are measured using a second set of surface electrodes, converting them into 2 impedance parameters, the resistance (R) and the reactance (X). The device has shown excellent reliability with intraclass correlations as high as 0.99 [31]. The full measurement of a particular muscle includes the following steps: 1) Applying saline to the surface of the skin using a saline wipe; 2) Placing the EIM sensor on the surface of the skin; and 3) Pressing a button on the device to begin a measurement. Each EIM measurement takes 2–5 s. Typically, each muscle is tested 3 times to make sure the measurement is repeatable. The following bilateral muscles will be tested: deltoid, biceps, triceps, vastus lateralis, tibialis anterior, and medial gastrocnemius.

### Motor function measure domain 1 (MFM1)

The MFM Domain 1 is a validated evaluator-administered functional measure for neuromuscular disorders, with 13 items related to standing position and transfers [68]. There are standard instructions for administration and it takes about 10 min to perform.

### Facial function

The IOPI is a means to quantify lip, tongue, and buccal strength using a validated tool with published ranges for normative data for lingual measurements [69]. For lip strength testing, the IOPI bulb is placed between the lips in midline and the subjects is instructed to squeeze the lips together as hard as he/she can for 3–5 s (repeated 3 times with a 30 s interval between trials). Endurance testing is conducted by asking the subject to hold 50% of the maximum lip press for as long as possible while viewing the light display on the IOPI to gauge 50% effort. Similar procedures are executed to assess anterior and posterior tongue and buccal strength and fatigue.

### Reachable workspace

Subjects are seated in front of a stereo-camera and perform a standardized upper extremity movement protocol under the supervision of a study clinical evaluator [70, 71]. Five hundred grams wrist weights are added. The standardized simple set of movements consist of lifting the arm from the resting position to above the head while keeping the elbow extended, performing the same movement in vertical planes at around 0, 45, 90, and 135 degrees. The second set of movements consists of horizontal sweeps at the level of the umbilicus and shoulder. Each set of movements is repeated three times for the left and right arms. A reduction in reachable workspace has previously been shown to reflect upper extremity strength impairment in FSHD [70].

### Strength testing

Strength testing will be performed using MMT, fixed QMT, and maximal isometric hand grip strength using a hand held force dynamometer [18, 49, 72]. For MMT a modified Medical Research Council 10 point scale will be used – with standardized positions for each muscle. QMT will be performed using a fixed myometry testing system, with a force transducer attached by an inelastic strap to a metal frame.

### Respiratory function

We will obtain forced vital capacity and forced expiratory volume in 1 s, both standardized outcomes used commonly in the clinic and in clinical trials.

### DEXA

Whole body and regional lean muscle mass will be measured via DEXA. DEXA uses a very small amount of x-ray energy (0.5 to 1.0 mREM vs. 20–30 mREM for a chest x-ray) to measure body composition. DEXA provides a practical approach to estimate lean muscle mass and has been utilized previously in clinical trials in neuromuscular disease [63, 73]. Female participants will have a pregnancy test performed prior to testing.

### Severity scores

A limited physical exam and strength testing (MMT) will be used to derive two FSHD clinical severity scores, the Clinical Severity Score (CSS) and the FSHD Clinical Score (FCS) [43, 44]. These severity scores both rank weakness in the face, shoulders, arms, distal, and proximal lower extremities on either a 10- (CSS) or 15-point scale (FCS). Both have been shown to be reliable, and have been used in genetic studies to identify mildly and severely affected FSHD individuals.

### Patient reported outcomes

The FSHD-HI questionnaire was designed to measure both overall FSHD health-related quality-of-life and 14 separate subdomains including: 1) mobility and ambulation; 2) fine motor and distal arm weakness; 3) proximal upper extremity and shoulder limitation; 4) trunk weakness; 5) FSHD-specific activity impairment; 6) emotional distress; 7) FSHD-specific impaired body image; 8) cognitive impairment; 9) social role dissatisfaction; 10) social role limitations; and FSHD-specific symptoms of: 11) fatigue; 12) pain; 13) communication difficulties/facial weakness; and 14) gustatory dysfunction. Each of these subdomains has been previously identified by FSHD patients as having the greatest importance to their specific population and is represented by a specific block of items [20, 50]. The PROMIS57 is an instrument developed by the NIH PROMIS initiative. It has been tested in general populations and generates scores for physical function, and the impact of physical limitations on daily life [45, 49]. The UEFI 15 is a validated patient reported measure for adults with upper extremity dysfunction [46, 47]. The FDI physical score is a short 5 item questionnaire that assesses the physical impact of facial weakness [48]. A fall and exercise questionnaire assesses average monthly falls and near falls, and average weekly amount of exercise (see Additional file 1). A fall assessment will be completed weekly for 3 months after the month 3 visit. Subjects will be asked to respond to an email every week, for 12 consecutive weeks, that asks about any falls they have had over the past week. The fall diary will be kept on a password-protected server where the subjects will sign in and complete the fall diary each week. A standard questionnaire will ask about the effect of FSHD on work/occupation.

### Anchor to determine minimal clinically important changes

Participants will be asked to complete a self-assessment “domain-delta” questionnaire at the 3- and 12-month visits, which is designed to accompany the FSHD-HI [20, 50]. The purpose of the “domain-delta” questionnaire is similar to that of a global rating of change questionnaire in that it will determine each patient’s perceived change in their health related quality-of-life between baseline and follow up. This questionnaire will inquire about total health as well as health related to 14 subdomains self-identified as important by patients during development of the FSHD-HI [20].

### Genetic testing

Genetic testing is a requirement for participation in the study. Detailed genetic testing includes accurate measurement of the D4Z4 repeat number on chromosome 4, determination of the A/B allele variants, and measurement of D4Z4 methylation levels [74]. In particular, for this study we will focus on the associations between D4Z4 repeat number and measures of disease progression. We will also evaluate the difference between the expected methylation based on D4Z4 repeat units alone, and the actual measured methylation values. With the assistance of prof. Silvère van der Maarel’s laboratory in Leiden University Medical Center, all patients in the study will have detailed genetic testing. Each subject will have approximately 25 mL of blood collected at baseline and month 3 for genetic testing. The 3-month sample will be stored for use as a back-up for any lost samples, or for use in future studies (e.g. genome-wide association studies). Two 2.5 ml PAX gene tubes will be collected at baseline. One will be sent to Leiden along with blood for genetic testing and the other stored locally at − 80 °C to be sent to the University of Rochester Medical Center (URMC) biorepository. An additional PAX gene tube will be collected at 3 months and will be sent to the URMC biorepository.

### Serum and plasma extraction

All sites will implement an identical serum and plasma collection protocol. At least one aliquot of serum from each of 3 visits will be stored centrally at URMC as part of an existing FSHD biorepository and the remaining aliquots will remain at the respective network site. The sera and plasma will be made available to researchers interested in studying serum biomarkers along with anonymized clinical information. Participants will have approximately 10 mL of blood collected at each visit for serum extraction and 10 mL of blood for plasma extraction.

### Data collection

During the conduct of the study, data collected will include the minimum necessary to address study aims: demographics, medical and medication histories, and documentation of physical and functional examination results. The study will utilize the infrastructure of the Muscle Study Group’s (MSG) Data Coordination Center (DCC) at URMC. The DCC will provide REDCap data entry training to all site staff and provide password protected access to the database for anonymized data entry.

### Sample size considerations

We will recruit 220 subjects with FSHD for this study, 160 from the US and 60 from European sites. It is expected that there will be approximately 165 subjects who complete evaluations at 18 months. A sample size of 165 subjects with data at 18 months will provide 90% power to detect a standardized response mean of 0.254 in any of our outcome variables using a paired t-test and a 5% significance level (two-tailed). By comparison, the estimated standardized response mean for the 12-month change in composite MMT score in our natural history study of FSHD was approximately − 0.30 [18]. Also, a sample size of 220 subjects at the baseline visit will provide at least 90% power to detect correlations between the new COAs (FSHD-COM and EIM) and existing outcomes of 0.22 or greater using a test with a 5% significance level (two-tailed). A sample size of 165 subjects will provide 90% power to detect correlations that are only slightly larger (0.25 or greater) between changes in outcomes over 18 months. Finally, a sample size of 220 subjects at baseline will provide > 90% power to detect that an intraclass correlation coefficient for test-retest reliability is significantly greater than a null hypothesis value of 0.70 (minimum acceptable) when the true value is 0.79 or higher, using a one-tailed test with a 5% significance level [75]. Within each center, a sample size of 20 subjects at baseline will provide > 80% power to detect that an intraclass correlation coefficient for test-retest reliability is significantly greater than a null hypothesis value of 0.70 (minimum acceptable) when the true value is 0.89 or higher, using a one-tailed test with a 5% significance level [75].

### Statistical considerations

#### Objective 1: reliability and validity

Test-retest reliability of the FSHD-COM and EIM measurements (components and composite scores), for each site and overall, will be quantified using intraclass correlation coefficients computed using one-way random effects models. Ninety-five percent lower confidence bounds will be computed for these quantities. Transformations will be attempted, if necessary, for outcomes that are not normally distributed. Bland-Altman plots will also be used for graphical examination of reliability [76]. The cross-sectional data obtained in 220 FSHD patients at baseline will be used to describe the sample and examine the relationships between the FSHD-COM and clinical severity scores (CSS, FCS), MFM, different measures of patient reported function (FSHD-HI and its subscales, PROMIS57, FDI, and UEFI), strength (QMT and MMT composite scores), reachable workspace, spirometry, IOPI, lean muscle mass, and D4Z4 deletion size. These bivariate associations will be examined using standard correlation and regression analyses. It is hypothesized that these associations will be moderate, but not so strong that one would consider the FSHD-COM redundant with the existing measures. A factor analysis will be performed to examine the structure of the FSHD-COM and determine whether the different components group together in a logical manner; Cronbach’s α will be used to assess the internal consistency of the scale.

For EIM, the metrics of primary interest include the reactance and phase at 50 kHz, 100 kHz, and the 50/200 kHz ratio. The data will be analyzed on individual muscles (or muscle groups) and on composite scores. Associations between EIM-derived measures and QMT measures in individual muscles (or muscle groups) will be examined. Associations between the composite EIM measure and the FSHD-COM, MFM, clinical severity scores, measures of patient-reported function, composite strength scores, reachable workspace, spirometry, lean muscle mass, and D4Z4 deletion size will also be examined using standard correlation and regression analyses.

Relationships between the new COAs and other variables such as age, gender, age at symptom onset, years since symptom onset, and years since diagnosis will be similarly examined, but these analyses will be more exploratory in nature since these associations are not necessarily expected to be strong.

Similar analyses will be performed to determine the associations between changes in the new COAs and changes in the other outcomes (clinical severity scores, measures of patient-reported function, composite strength scores, lean muscle mass). Associations will be examined using the changes from baseline to 12 and 18 months; the associations among 18-month changes are expected to be stronger than those among 12-month changes. In addition, it will be of particular interest to examine the associations between 3-month changes in EIM outcomes and 12- and 18-month changes in other outcomes (including EIM) to see if there is support for the use of EIM as a predictive marker in short-term early-phase clinical trials.

#### Objective 2: responsiveness to change over time and MCIC

Responsiveness of the outcome measures to change over 12–18 months will be assessed. This is reasonable under the assumption that measurable progression occurs in FSHD over a period of 1 year, as previously shown [18]. Paired t-tests will be used to test the null hypothesis of zero mean change at both 12 and 18 months for each measure.

Various statistics can be used for quantifying responsiveness, and the *effect size* and *standardized response mean* have been most highly recommended for this purpose [77]. The *effect size* is defined as the mean change divided by the standard deviation of the baseline value [78]. The *standardized response mean* is defined as the mean change divided by the standard deviation of the changes from baseline [79]. For within-group (paired) comparisons, as is the case here, the standardized response mean is equivalent to the paired t-test (the two differ only by a factor of the square root of the sample size). The bootstrap resampling technique will be used to perform formal statistical comparisons between different outcome measures in terms of these two measures of responsiveness [80].

Anchor-based and distribution-based methods will be used to determine the minimal clinically important changes (MCICs) on the FSHD-COM [81]. Mean responses on the FSHD-COM will be described for each of the categories of the “domain delta” questionnaire (e.g., unchanged, a little better, a lot better, etc.). Receiver operating characteristic (ROC) curve methods will be used to select a cut-off for the 12-month change in the FSHD-COM that is best at minimizing misclassification error, i.e., best distinguishes those who indicate that they are at least “a little better” on the “domain-delta” questionnaire and those who indicate otherwise. The 12-month changes in the FSHD-COM that correspond to effect sizes ranging from 0.30 to 0.50 standard deviation units will also be described and compared to the MCIC identified by ROC curve methods. These analyses will be repeated for 18-month changes and for other outcome measures. Anchor-based and distribution-based methods are well known to have their strengths and limitations and examination of the results derived by both methods will be useful in reaching consensus on recommendations in this regard for future trials in FSHD [81].

*Objective 3: Baseline correlates of change over time*. Identification of factors contributing to the variability of disease progression may help in the design of future clinical trials. The outcomes of primary interest are the changes from baseline to 12 and 18 months in the FSHD-COM. The baseline variables of primary interest include the FSHD-COM score, the composite EIM measure, clinical severity scores, measures of patient-reported function, composite strength scores, lean muscle mass, age, gender, age at symptom onset, years since symptom onset, and D4Z4 deletion size. A multiple regression model will be constructed, and competing models will be evaluated using a best-subsets regression technique, in conjunction with Akaike information criterion and the Bayesian information criterion [82]. This information will be combined with clinical judgment to arrive at a final model.

Another set of exploratory analyses will be performed using regression trees to attempt to identify subgroups of FSHD subjects who have different rates of progression. Regression trees use recursive partitioning to partition the sample into different subsets that have different levels of mean change in the FSHD-COM [83]. A strength of these methods is that a cross-validation procedure is available for checking the final tree, which can be pruned back to avoid over-fitting.

## Discussion

At its completion, ReSolve will be the largest international, prospective, observational study in FSHD. In addition to validating two novel COAs, the study will provide a better understanding of functional, genetic, and demographic correlates of disease progression. This information is critical for selecting valid and reliable outcome measures, sample size calculations, specifying rational eligibility criteria, and the efficient conduct of future clinical trials.

There are limitations to this study. Our sample of FSHD patients may not be representative of the entire population. For example, patients with significant disability will be underrepresented. We have excluded those that are non-ambulatory since these patients will likely be excluded in future clinical trials and so that all included patients will be able to complete every assessment. In addition, as a multi-site study with many investigators and clinical evaluators, reliability is a concern. To mitigate the magnitude of this problem, we have implemented a training protocol similar to that used in industry. Still, it is important to point out that these are similar problems that would be faced in future clinical trials.

This study also has several unique aspects that are important to mention. This is the first study conducted using the recently established FSHD-CTRN. With support from industry, FSHD researchers, and patients, the network was formed as a broad collaborative effort to find treatments for FSHD. In keeping with this mission, continued collaboration and data sharing is essential. The data from this study will be made available for any investigator or company pursuing treatments for FSHD. Additionally, the creation of a biorepository as part of this study provides an invaluable resource for future research. With this network in place, another goal is to build a strong partnership with patients and their families. This is being accomplished by increasing patient engagement in all aspects of the clinical trial process. Patients were involved in defining what is clinically meaningful to them as well as in addressing issues related to subject recruitment and retention. Overall, we hope to leverage the strengths of the network and patient engagement in order to hasten drug development. Specifically, this study will help to address the challenges in FSHD clinical trial preparedness due to FSHD’s slow disease progression and lack of current biomarkers that clearly correlate with disease activity and severity.

## Additional file

Additional file 1: **Table S1** FSHD-CTRN ReSolve Investigators (DOCX 21 kb)

## Data Availability

Not applicable

## Abbreviations

6MWT: 6 minute walk test
ASO: Antisense oligonucleotide
CE: Clinical evaluator
COA: Clinical outcome assessment
CSS: Clinical severity score
CTSA: Clinical and Translational Science Awards
DCC: Data coordination center
DEXA: Dual-energy X-ray absorptiometry
DMD: Duchenne muscular dystrophy
*DUX4*: Double homeobox 4
EIM: Electrical impedance myography
FCS: FSHD clinical Score
FDI: Facial disability index
FSHD: Facioscapulohumeral dystrophy
FSHD-COM: FSHD composite outcome measure
FSHD-CTRN: FSHD Clinical Trial Research Network
FSHD-HI: FSHD-health Index
IOPI: Iowa oral performance instrument
MCIC: Minimal clinically important change
MFM: Motor function measure
MMT: Manual muscle testing
MSG: Muscle Study Group
QMT: Quantitative myometry
ROC: Receiver operating characteristic
TUG: Timed up and go
UEFI: Upper extremity functional index
URMC: University of Rochester Medical Center
