# Supplementary material for: Clinical trial readiness to solve barriers to drug development in FSHD (ReSolve): protocol of a large, international, multi-center prospective study
Source: BMC Neurol. 2019 Sep 10;19:224. doi: 10.1186/s12883-019-1452-x (PMC6734593; doi:10.1186/s12883-019-1452-x)
Supplement: Supplementary file 1 — Table S1 FSHD-CTRN ReSolve Investigators (DOCX 21 kb) [file 12883_2019_1452_MOESM1_ESM.docx]

Additional file 1: Table S1: FSHD-CTRN ReSolve Investigators

| **Site** | **Personnel** | **Role** |
| --- | --- | --- |
| **University of Kansas Medical Center, Kansas City, KS** | Jeffrey Statland, MD | Principal Investigator |
|  | Mazen Dimachkie, MD | Co-Investigator |
|  | Mamatha Pasnoor, MD | Co-Investigator |
|  | Kiley Higgs, BSBA, CCRP | Project Manager |
|  | Katherine Roath, MS, CCRP | Clinical Research Coordinator |
|  | Ayla McCalley, CCRP | Clinical Research Coordinator |
|  | Melissa Currence, PTA, BA | Clinical Evaluator |
|  | Laura Herbelin, BS, CCRP | Clinical Evaluator |
| **University of Rochester Medical Center, Rochester, NY** | Rabi Tawil, MD | Principal Investigator |
|  | Johanna Hamel, MD | Co-Investigator |
|  | Leann Lewis, MS | Clinical Research Coordinator |
|  | Katy Eichinger, PT, PhD, DPT, NCS | Clinical Evaluator |
| **Ohio State University Wexner Medical Center, Columbus, OH** | Samantha LoRusso, MD | Principal Investigator |
|  | W. David Arnold, MD | Co-Investigator |
|  | Tabitha Alexander | Clinical Research Coordinator |
|  | Matthew Yankie, PT, DPT, ATP | Clinical Evaluator |
|  | Kristina Kelly, PT, DPT, NCS, EdM | Clinical Evaluator |
| **Virginia Commonwealth University, Richmond, VA** | Nicholas Johnson, MD, MS-CI | Principal Investigator |
|  | Brittney Holmberg | Clinical Research Coordinator |
|  | Liz Diaz | Clinical Research Coordinator |
|  | Aileen Jones, PT, DPT | Clinical Evaluator |
|  | Amanda Butler, PT, DPT | Clinical Evaluator |
| **University of Utah, Salt Lake City, UT** | Russell J Butterfield, MD, PhD | Principal Investigator |
|  | Sarah Moldt, BS | Clinical Research Coordinator |
|  | Amelia Wilson, PT, DPT | Clinical Evaluator |
|  | Melissa McIntyre, PT, DPT | Clinical Evaluator |
| **Kennedy Krieger Institute, Baltimore, MD** | Kathryn Wagner, MD, PhD | Principal Investigator |
|  | Doris Leung, MD, PhD | Co-Investigator |
|  | Genila Bibat, MD | Clinical Research Coordinator |
|  | Mary Yep BS,CCRP | Clinical Research Coordinator |
|  | Nikia Stinson, PT | Clinical Evaluator |
|  | Andrea Jaworek, PT, DPT ,CLT | Clinical Evaluator |
| **University of Washington, Seattle, WA** | Leo Wang, MD, PhD | Principal Investigator |
|  | Laura Sissons-Ross | Clinical Research Coordinator |
|  | Laura Johnstone, PT | Clinical Evaluator |
| **University of California, Los Angeles, Los Angeles, CA** | Perry Shieh, MD, PhD | Principal Investigator |
|  | Christy Skura, PT, DPT, PCS | Clinical Evaluator |
|  | Dianne DeGuzman, BS | Clinical Research Coordinator |
| **Radboud University Medical Center, Nijmegen, the Netherlands** | Karlien Mul, MD, PhD | Principal Investigator |
|  | Yvonne Cornelissen | Clinical Evaluator |
| **Centre Hospitalier Universitaire de Nice, Nice, France** | Sabrina Sacconi, MD, PhD | Principal Investigator |
|  | Luisa Villa | Co-Investigator |
|  | Angela Puma | Co-Investigator |
|  | Manuela Gambella, PhD | Clinical Research Coordinator |
|  | Ying Shi | Clinical Research Coordinator |
|  | Jeremy Garcia | Clinical Evaluator |
| **University of Milan, Milan, Italy** | Valeria A Sansone, MD, PhD | Principal Investigator |
|  | Elena Carraro, MD | Co-Investigator |
|  | Fatmira Beshiri, PT | Clinical Evaluator |
|  | Luca Mauro | Data Coordinator |
